# Structural Biology of Influenza Hemagglutinin: An Amaranthine Adventure

**DOI:** 10.3390/v12091053

**Published:** 2020-09-22

**Authors:** Nicholas C. Wu, Ian A. Wilson

**Affiliations:** 1 Department of Biochemistry, University of Illinois at Urbana-Champaign, Urbana, IL 61801, USA; nicwu@scripps.edu; 2 Carl R. Woese Institute for Genomic Biology, University of Illinois at Urbana-Champaign, Urbana, IL 61801, USA; 3 Department of Integrative Structural and Computational Biology, The Scripps Research Institute, La Jolla, CA 92037, USA; 4 The Skaggs Institute for Chemical Biology, The Scripps Research Institute, La Jolla, CA 92037, USA

**Keywords:** influenza virus, hemagglutinin, evolution, host receptor binding, sialylated glycans, membrane fusion, antibody, antigenicity, vaccine, escape mutations

## Abstract

Hemagglutinin (HA) glycoprotein is an important focus of influenza research due to its role in antigenic drift and shift, as well as its receptor binding and membrane fusion functions, which are indispensable for viral entry. Over the past four decades, X-ray crystallography has greatly facilitated our understanding of HA receptor binding, membrane fusion, and antigenicity. The recent advances in cryo-EM have further deepened our comprehension of HA biology. Since influenza HA constantly evolves in natural circulating strains, there are always new questions to be answered. The incessant accumulation of knowledge on the structural biology of HA over several decades has also facilitated the design and development of novel therapeutics and vaccines. This review describes the current status of the field of HA structural biology, how we got here, and what the next steps might be.

## 1. Introduction

Four types of influenza virus, A, B, C, and D, are known. Influenza A and B viruses can cause severe symptoms and mortality in the human population, whereas influenza C virus only manifests itself in mild disease and influenza D virus does not circulate in humans. A major difference between influenza A and B viruses is that influenza B virus is almost exclusively observed in humans, whereas influenza A virus has a diverse and extensive reservoir in aquatic birds that occasionally spills over to humans directly or via domestic animals, such as pigs, as new pandemics or emerging viruses [1]. As a result, influenza A viruses receive much more attention than other influenza types even though influenza A and B both co-circulate in the human population as seasonal viruses. Influenza A virus can be further divided into subtypes based on the antigenicity of the surface glycoproteins hemagglutinin (HA) and neuraminidase (NA), with 18 known subtypes of HA (H1–H18) and 11 subtypes of NA (N1–N11). Similar to influenza A virus, influenza B virus also has two surface glycoproteins HA and NA, which diverged into two lineages, Victoria and Yamagata, during the 1980s [2]. In contrast, influenza C and D viruses only have one surface glycoprotein hemagglutinin-esterase fusion (HEF) [3] that encompasses both HA and NA activities. Four known influenza A pandemics have been documented in human history, namely 1918 Spanish flu (H1N1), 1957 Asian flu (H2N2), 1968 Hong Kong flu (H3N2), and 2009 swine flu (H1N1), although others undoubtedly have occurred prior to these [4]. Occasionally, other influenza A subtypes, such as H5N1, H5N6, H6N1, H7N7, H7N9, H9N2, and H10N8, also infect humans through cross-species transmission but so far lack the ability for human–human transmission. Nevertheless, zoonotic influenza subtypes can be highly pathogenic, with a mortality rate of up to 60% in hospitalized patients [5]. Currently, vaccines (trivalent and quadrivalent) are available against seasonal influenza viruses, including subtypes H1N1 and H3N2 of influenza A virus and for the two lineages of influenza B virus [6]. However, the effectiveness of seasonal influenza vaccine is often quite low, especially against H3N2 viruses, despite the vaccine components being updated annually [7]. Therefore, influenza vaccine development remains an active research area.

Since HA is essential for viral entry through engaging the host receptor and mediating membrane fusion, many anti-HA antibodies are neutralizing. As a result, HA has been the major target for influenza vaccine development, although NA has recently been “rediscovered” as an attractive target [8]. HA exists as a homotrimer with a highly variable globular head domain, which contains the receptor-binding site (RBS), atop a conserved stem domain, which houses the fusion machinery. The first HA structure was reported in 1981 [9]. Since then, structural biology of HA has provided many important insights into its receptor-binding function, fusion mechanism, antigenicity, and genetic plasticity, which in turn has facilitated the design and development of therapeutics and vaccines against influenza virus. Here, we review some of the noteworthy developments in the field of influenza HA structure biology and highlight questions that remain to be addressed. H3 numbering is used in this review unless otherwise stated.

## 2. Receptor Binding of Influenza HA

Back in the early 1940s, George Hirst reported the ability of influenza virus to agglutinate chicken red blood cells (RBCs) [10] and attributed this to adsorption of the HA onto the RBCs [11]. In the late 1940s, an enzyme from *Vibrio cholerae* was discovered with the ability to prevent influenza virus from agglutinating red blood cells [12,13]. Subsequent identification of the enzymatic product revealed sialic acid as the receptor of influenza virus [14]. However, the location and molecular characteristics of the RBS were unclear until the first HA structure was determined in 1981 [9]. The RBS was identified partly due to its sequence conservation, structural resemblance to the wheat-germ agglutinin sialic acid-binding site [15], and from mutations that affect receptor specificity [16]. The first structure of HA in complex with sialic acid in 1988 confirmed the location of the RBS and sialic acid as the host receptor of influenza virus [17]. The RBS of influenza A HA is composed of four structural elements, 130-loop, 150-loop, 190-helix, and 220-loop, which are named after their positions on the primary amino acid sequence. Similarly, RBS of influenza B HA is composed of the 140-loop, 190-helix, and 240-loop, which are structurally equivalent to the 130-loop, 150-loop, and 190-helix in influenza A HA [18]. Four residues in the RBS are highly conserved across influenza A and B HAs: Trp153, His183, Leu194, and Tyr195 (H3 numbering, i.e., Trp158, His191, Leu201, and Tyr202 in influenza B numbering) [18]. However, the RBS of influenza and B also have important differences. For example, while Phe98 (H3 numbering, i.e., residue 95 in influenza B numbering) is highly conserved in influenza B virus [18], influenza A virus has a highly conserved Tyr at residue 98. In influenza A virus, the Y98F mutant has very poor receptor binding [19]. Although some animal influenza A viruses use N-glycolyl-neuraminic acid (NeuGc) [20], most influenza A and B viruses use N-acetyl-neuraminic acid (Neu5Ac) as a receptor [21], whereas influenza C virus mainly uses N-acetyl-9-*O*-acetylneuraminic acid (Neu5,9Ac_2_) [22,23]. In comparison, influenza D virus seems to be able to tolerate the broadest range of sialic acid modifications on the host receptor, likely due to its more open receptor-binding cavity [24].

Influenza A viruses isolated from humans and avian species have different types of receptor specificity [25]—human influenza viruses preferentially recognize α2,6-linked sialic acids (human-type receptors), whereas avian influenza viruses preferentially recognize α2,3-linked sialic acids (avian-type receptors). When binding to HA, the human-type receptor displays a folded-back configuration, whereas avian-type receptors display a more extended configuration [26]. This HA receptor specificity switch appears to be a molecular requirement for new influenza pandemics. However, the mutations that are responsible for receptor specificity switch are subtype-dependent. H1N1 pandemics in 1918 and 2009 both acquired a pair of mutations E190D/G225D in order to switch receptor specificity from avian-type to human-type receptors [27,28,29,30,31], whereas the H2N2 pandemic in 1957 and H3N2 pandemic in 1968 acquired another pair of mutations, Q226L/G228S, to do so [16,32,33,34]. Furthermore, studies have shown that H9N2 viruses with a naturally occurring mutation Q226L display human virus-like cell tropisms, raising some questions about pandemic potential [35,36]. While successful switch of receptor specificity has not been observed for most naturally occurring subtypes, adaptation experiments and mutagenesis studies in the lab have demonstrated the feasibility of H4 [37], H5 [38,39], H6 [40], H7 [41], and H10 [42] subtypes being able to acquire human-type receptor specificity, although some have more complex mutational trajectories than for H1N1, H2N2, and H3N2. Structural analyses demonstrated that stabilization of the interaction with α2,6-linked sialic acid in the folded-back configuration is often the key for switching receptor specificity to human-type receptors across different subtypes [16,31,34,37,40,41,42,43,44].

**Figure 1:**
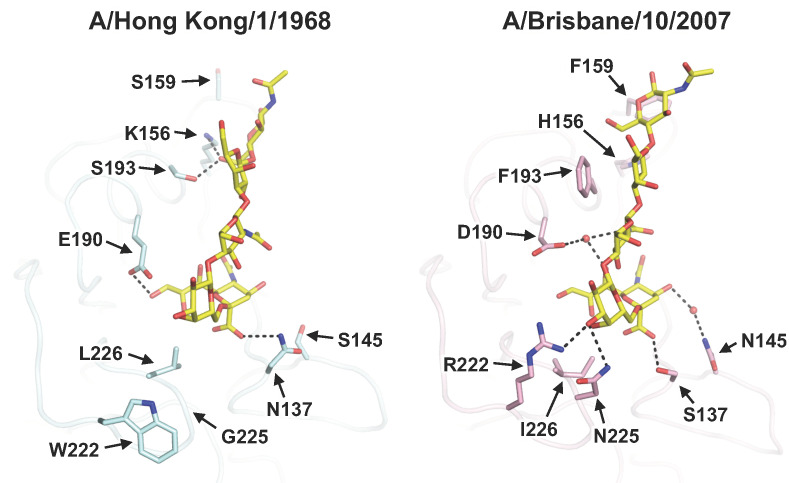
Natural evolution of receptor-binding mode in seasonal influenza virus. Crystal structures of human receptor analog (6′-SLNLN, yellow) in complex with HAs from two human H3N2 influenza strains that were isolated 40 years apart, namely A/Hong Kong/1/1968 (cyan) and A/Brisbane/10/2007 (pink), are shown. Representative residues in the receptor binding site (RBS) that were mutated during the course of natural evolution are shown in stick representations. Hydrogen bonds are shown as dashed lines. All structure images in this review were rendered by PyMOL (www.pymol.org).

Receptor specificity can also continue to evolve when seasonal viruses circulate in the human population, due to natural mutations that are likely a response to immune selection pressure. This phenomenon has recently been reported in human H3N2 viruses, which have evolved a preference for long, branched sialylated glycans with extended poly-N-acetyl-lactosamine (poly-LacNAc) [45]. Structural studies have also shown that the receptor-binding mode of human H3N2 HA has changed over time and is associated with mutations in all four structural elements of the RBS [46,47,48,49]. Specifically, natural mutations in the 130-loop and 220-loop have introduced additional hydrogen bonds (H-bonds) to Sia-1 and Gal-2 of the receptor [47,48,49], whereas a number of other mutations in the 190-helix and 150-loop allow the subsequent sugar moieties (GlcNAc-3, Gal-4, and GlcNAc-5) to move closer to the RBS [47,48,49] (Figure 1). Such changes in receptor binding can alter the amino acid preference at particular positions in the RBS [47,48]. For example, residue 190 in the RBS of H3N2 HA was almost exclusively Glu before the 1992–1993 influenza season but mutated to Asp in almost all strains from then on. Interestingly, reverting the Asp back to Glu in strains from year 2007 onward abolished receptor binding, due to the concomitant change in the receptor-binding mode [47]. In fact, evolutionary contingency, which describes sequence variants that were previously fit but then become unfit and extinct, as well as evolutionary entrenchment, which describes sequence variants that were previously unfit and then become fit and emerge, are common in the HA RBS of human H3N2 viruses [48]. As seasonal influenza viruses continue to evolve in the human population, it will be fascinating to observe how the receptor-binding mode is able to change (or not) in the future, which would allow the H3N2 virus to continue its over 50 years of sustained circulation in the human population.

Interestingly, bat influenza A viruses H17N10 and H18N11 do not utilize sialylated glycans as receptors [50,51]. Crystal structures of the HA from H17N10 and H18N11 viruses indicate that their RBS is highly acidic, which would electrostatically repulse sialic acid and hence would have substantially different biochemical properties from the other HA subtypes (i.e., H1–H16) even although the overall architecture of the RBS is roughly similar [50,51]. Recent studies have revealed that major histocompatibility complex class II (MHC-II) human leukocyte antigen DR isotype (HLA-DR) can act as a receptor for bat influenza A viruses [52,53]. However, a structure of the complex between bat influenza HA and HLA-DR has not been reported. Therefore, the receptor-binding mechanism of bat influenza HA remains elusive. Influenza viruses have also been discovered in species as diverse as eel, toad, and hagfish using a meta-transcriptomic approach [54]. Nonetheless, the HAs from these influenza viruses have not been functionally characterized and their receptors are currently unknown. Additional influenza virus subtypes as well as types will likely be discovered in the future, and it will be to interesting to see whether other host receptors are employed.

## 3. HA Fusion Machinery and Mechanism

After attaching to the host receptor, endocytosis transports the influenza viral particle to the endosome, where the pH becomes acidic. The acidic pH triggers viral–host membrane fusion that is mediated by conformational rearrangements in the HA. The prerequisite for such conformational rearrangements is proteolytic processing of the HA. HA is translated as a single polypeptide chain HA0, which is then cleaved by host proteases into the HA1 and HA2 subunits. The membrane fusion machinery is encoded mainly by HA2, while HA1 is entirely responsible for receptor binding, as outlined in the previous section. The overall structure of uncleaved HA0 is almost identical to the cleaved HA [55]. The cleavage site on HA0 is presented as a surface loop on the HA stem, which is proximal to the viral membrane compared to the HA head. The amino acid sequence at the cleavage site is a well-characterized pathogenic factor [56]. While most influenza A strains carry a monobasic cleavage site, some highly pathogenic avian influenza A strains carry a polybasic cleavage site that can be processed by ubiquitously expressed furin. Upon cleavage, the C-terminus of HA1 remains solvent exposed, whereas the N-terminal of HA2, which represents the hydrophobic fusion peptide, inserts into a buried cavity that is composed of ionizable residues including HA1 His17, as well as HA2 Asp109, Asp112, and Lys117 [55]. This metastable conformation is then poised for low pH-induced structural rearrangements to accomplish viral–host membrane fusion.

**Figure 2:**
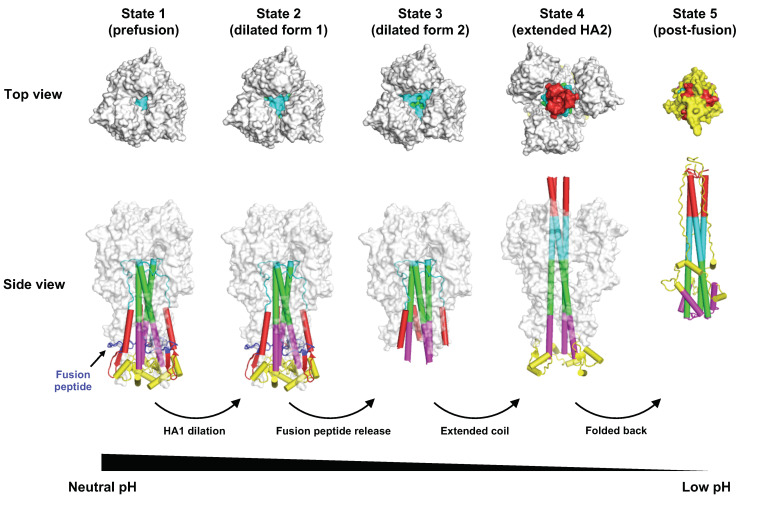
Conformational change of HA during pH-induced membrane fusion. Different intermediates states of HA during pH-induced conformational change were identified by cryo-EM [60]. The top and side views of state 1 (prefusion conformation, PDB 6Y5H) [60], state 2 (dilated form 1, PDB 6Y5I) [60], state 3 (dilated form 2, PDB 6Y5J) [60], state 4 (extended HA2, PDB 6Y5K) [60], and state 5 (post-fusion conformation, PDB 1QU1) are shown [61]. Of note, after fusion peptide is released from state 2, the fusion peptide becomes disordered [60]. In state 3, the membrane proximal region (yellow) is also disordered [60]. Different components in the HA2 that are involved in structural rearrangements between pre- and post-fusion structures are in different colors.

In fact, the ability of HA to undergo pH-dependent structural rearrangement has been known since the early 1980s based on circular dichroism, electron microscopy, and sedimentation analyses [57]. Subsequent analyses demonstrated that, after conformational changes, HA is susceptible to trypsin digestion, where HA1 residues 28 to 328 (globular domain) are released, while HA1 residues 1 to 27 remain covalently linked through a disulfide bond to the intact HA2 subunit [57,58]. A crystal structure of this trypsin-digested product containing the intact HA2 subunit, which represents the post-fusion conformation of HA, was reported in the mid-1990s [59]. The post-fusion conformation of HA2 features substantial rearrangements of helices and connecting segments to form a 100 Å-long α-helix in each protomer, which assemble as a three-stranded coiled coil at trimer interface (Figure 2). In addition, the hydrophobic fusion peptide relocates to the top of the helix ready for membrane insertion.

Structures of intermediates during the HA fusion process have been probed by low-resolution cryo-electron microscopy (cryo-EM) [62,63], as well as X-ray crystallography [64]. Nonetheless, a more complete picture of HA fusion intermediate structures was described only recently [60], by taking advantage of advances in high-resolution cryo-EM [65]. Specifically, after incubation of HA at low pH for different times (10 s, 20 s, 60 s, and 30 min), HA conformational changes were examined by cryo-EM [60] (Figure 2). Three-dimensional (3D) classification and reconstruction at different time points revealed three sequential intermediate forms of HA, including one with a 150 Å-long three-stranded α-helix coiled coil [60]. However, it is still unclear how the lipid bilayer membranes from the virus and host are fused together because most structural studies use the HA ectodomain and membranes are often excluded. The feasibility of structurally characterizing full-length HA, which includes the transmembrane region, has also recently been demonstrated by cryo-EM [66]. Therefore, future studies should be able to explore the conformational changes during influenza virus–host membrane fusion in the context of full-length HA and in the presence of a membrane.

## 4. Antibodies to Influenza HA

**Figure 3:**
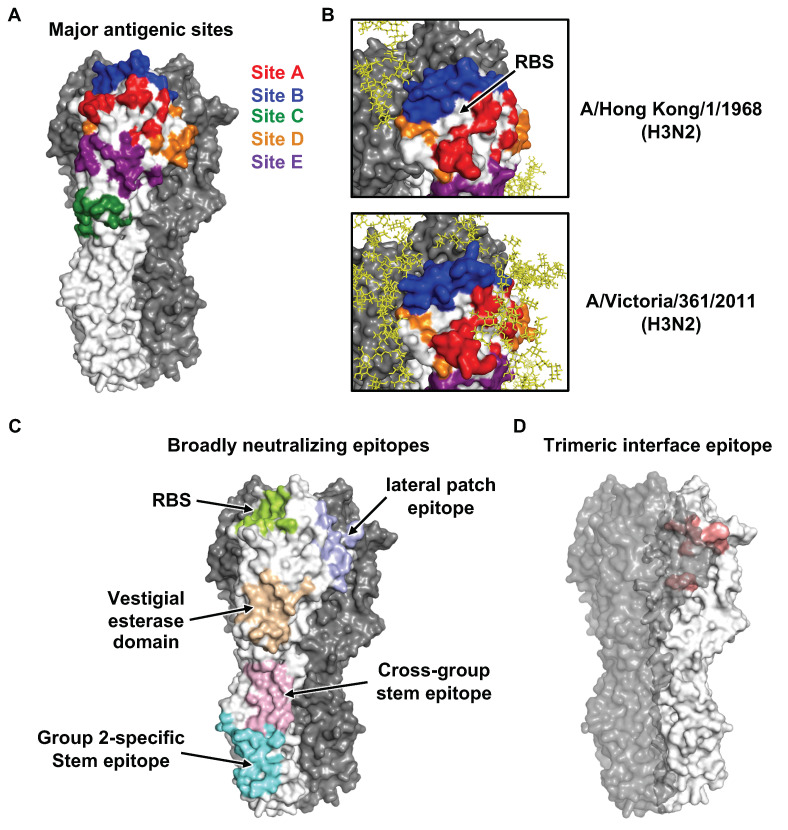
Conventional antigenic sites and recently identified epitopes. (**A**) The five major antigenic sites A-E on H3N2 HA are shown. (**B**) There is an accumulation of glycosylation sites during human H3N2 evolution. While many antigenic sites have now been masked by glycans (yellow), antigenic site B (blue) remains exposed due to its proximity to the RBS, making it immunodominant in recent human H3N2 strains [95,96]. (**C**) Broadly neutralizing epitopes that have been identified in the past decade are shown. (**D**) A recently identified trimeric interface epitope is illustrated.

Based on analysis of the first HA structure [9] with known natural antigenic variants and laboratory escape mutants at the time [67,68,69,70,71,72,73,74], four major antigenic sites (A-D) in the H3 HA were identified and reported in a back-to-back paper with the HA structure in 1981 [9,75]. In the 1980s, a fifth antigenic site (E) was also identified [76,77]. Similarly, five major antigenic sites, namely Sa, Sb, Ca1, Ca2, and Cb, were identified in H1 HA during the early 1980s [78,79]. All of the major antigenic sites in H1 and H3 HAs as well as influenza B HA [80,81] are located in the HA1 globular head domain and their immunodominance can change over natural evolution (Figure 3A,B). However, the first structure of an antibody (HC19) in complex with HA was not reported until 1995 [82]. Antibody HC19 targets the RBS, which explains its neutralization activity. However, HC19 also recognizes RBS-proximal regions, which are highly variable across strains. As a result, escape mutants to HC19 could be readily identified. Consistently, subsequent studies demonstrated that major antigenic drift in seasonal influenza viruses is mostly driven by mutations within or near the RBS [83,84]. It is therefore not surprising that some of the mutations that arise during natural evolution of human influenza virus can alter both HA antigenicity and receptor binding [47,48,85,86]. Furthermore, egg-based seasonal influenza vaccines often carry egg-adaptive mutations in the HA RBS that allow the vaccine strain to bind to α2–3 linkage sialylated glycans on the chorioallantoic membrane but can also alter the antigenicity of HA, thereby decreasing vaccine effectiveness [49,87,88,89]. For example, one of the egg-adaptive mutations T160K would abolish an N-glycosylation site at N158 and appears to contribute to the poor seasonal influenza vaccine effectiveness in the 2016–2017 influenza season. In fact, accumulation of N-glycosylation in the HA1 globular head domain plays an important role in the antigenic drift of seasonal influenza virus [76,90,91]. A recent study has shown that N-glycosylation sites are added to HAs of seasonal influenza virus at discrete 5-to-7-year intervals, with an upper limit of ~6 and ~8 glycans in the HA1 globular head domains of H1N1 and H3N2, respectively [92]. The glycan form, occupancy, and heterogeneity at each N-glycosylation site on HA can be probed by mass spectrometry [93,94]. Moreover, some of the N-glycans on HA can be observed by X-ray crystallography and cryo-EM [9,64].

As compared to the variable globular head domain in HA1, the stem domain in HA2 is much more conserved. It had long been thought that neutralizing antibodies (nAbs) do not target the stem domain until the discovery of a mouse HA stem antibody C179 in 1993 [97]. Nevertheless, this observation was largely unappreciated and stem antibodies were not found in humans until the late 2000s [98,99,100] (Figure 3C). In the subsequent decade, many stem neutralizing and protective antibodies have been isolated and structurally characterized. Unlike neutralizing antibodies to the HA head, which generally block receptor binding, stem antibodies typically protect by interfering with the fusion machinery [101,102,103]. Due to high sequence conservation of the stem domain, stem antibodies usually exhibit higher breadth (i.e., broadly neutralizing antibodies, bnAbs) and interact with a greater range of influenza subtypes and strains compared to nAbs to the HA head. Recurring molecular features are observed in stem Abs. For example, the IGHV1-69 antibody heavy-chain germline gene is commonly used by the immune system for generation of stem antibodies due to the presence of a germline-encoded IFY motif, which can engage three highly conserved hydrophobic pockets in the HA stem region [98,100,101,102,104,105,106]. In addition, IGHD3-9, one of the heavy-chain diversity genes that encodes for an important part of complementarity determining region 3 of the heavy chain (CDR H3), is also utilized in many stem antibodies. The IGHD3-9 gene encodes an LXYFXWL motif that makes favorable interactions with four hydrophobic pockets in the HA stem. However, the breadth of some HA stem antibodies is often restricted to group 1 HAs (H1, H2, H5, H6, H8, H9, H11, H12, H13, and H16), since a conserved N-glycan at HA1 residue 38 in most of the group 2 HAs (H3, H4, H7, H10, H14, and H15) can sterically hinder accessibility to the HA stem epitope [102,107]. A few select IGHV1-69-encoded stem antibodies can manage to navigate around the N-glycan at HA1 residue 38 to achieve cross-group neutralization [101,104]. Some group 2-specific Abs bind to an epitope that is in the lower part of the stem domain and closer to the viral membrane (Figure 3C) and hence can avoid a clash with the N-glycan at HA1 residue 38 [108,109]. More recently, IGHV6-1 was found to be a germline gene that is often utilized in cross-group stem Abs [110,111,112,113]. Interestingly, the ancestral precursors of IGHV6-1-encoded cross-group stem Abs can be either group 1- or group 2-specific, depending on the CDR H3 sequences and conformations [113].

Over the past decade, several cross-group bnAbs that target HA RBS have also been discovered and characterized [114,115,116,117,118,119,120] (Figure 3C). While the reactivity of some RBS bnAbs is mostly limited to a particular subtype [121,122,123,124,125,126], they are still considered as broadly neutralizing in the sense of covering most if not all strains within a subtype (e.g., 5J8 [121] and CH65 [124] to H1 HA, as well as F045-092 [115] and 019-10117-3C06 [120] to H3 HA). Such antibodies could be very useful in protecting against antigenic drift in seasonal viruses, for example. In addition, subtype-specific bnAbs that target the vestigial esterase subdomain [127,128], “lateral patch” epitope on HA1 [129], and the junction between the ectodomain and membrane anchor have also been identified [66]. In 2019, an H7-specific bnAb was shown to target an epitope that partly involves the HA protomer–protomer interface in HA1 [130]. Such a finding demonstrated that an antibody epitope does not need to be completely solvent exposed in the prefusion conformation. In the same year, three other papers have reported an epitope that is almost exclusively in the HA protomer–protomer interface in HA1 [131,132,133] (Figure 3D). Some interface-targeting antibodies can cross-react with all influenza A subtypes and confer in vivo protection despite the lack of neutralization activity [131,132]. Therefore, it is now quite clear that ‘‘breathing’’ of the HA trimer [134] can allow antibodies to access cryptic epitopes that are transiently exposed and were not originally thought to be accessible in the HA prefusion conformation.

## 5. HA-Based Therapeutic and Vaccine Design

During the early 1990s, structural-based computational screening of around 55,000 small molecules resulted in the identification of benzoquinones and hydroquinones as HA fusion inhibitors [135]. One of the compounds, tert-butyl hydroquinone (TBHQ), had its binding mode to HA reported in 2008 [136]. TBHQ binds to a hydrophobic pocket in an interface region between HA monomers, which in turn stabilizes the HA prefusion conformation and prevents the conformational changes required for membrane fusion. Interestingly, Arbidol, which was developed as a general antiviral medication in Russia especially for respiratory diseases during the late 1980s [137], was more recently shown to inhibit HA-mediated membrane fusion by stabilizing the prefusion conformation [138,139]. In 2017, the structure of Arbidol in complex with HA revealed that the Arbidol binds to a similar location in the HA stem as TBHQ, but its binding site is much larger and more complex [140]. Structure-based optimization of Arbidol resulted in a compound with its affinity improved by two to three orders of magnitude, although it manifested low stability [141]. Thus, the stem region on HA that is targeted by both Arbidol and TBHQ represents a promising target for future influenza antiviral development.

Over the past decade, structural characterization of bnAbs to the HA have stimulated antiviral design, ranging from small protein binders [142,143,144,145] to peptides [146] to small molecules [147,148]. In addition, the discovery and characterization of bnAbs to HA have reignited aspirations and novel approaches towards a more universal influenza vaccine [149]. While universal influenza vaccine design has largely been focused on the stem domain [150,151,152,153,154], our recent study demonstrated the need to consider the potential for escape mutations to stem bnAbs, which can more rapidly emerge in the H3 subtype compared to other subtypes, such as H1N1 [155]. Indeed, some escape mutations have already been observed in low frequency in naturally circulating strains. To escape stem bnAbs, mutations can either decrease binding of stem bnAbs or enhance HA fusion ability [156]. Similarly, escape mutants to RBS bnAbs can be even more readily isolated [157,158]. Notwithstanding, studies in Zika virus, Ebola virus, HBV, and SARS-CoV-2 have shown that use of a well-designed antibody cocktail can minimize the emergence of escape mutants [159,160,161,162]. Thus, a universal influenza vaccine may need to induce a polyclonal response that targets both the RBS and stem domain to prevent or mitigate against escape. The advantage of simultaneously targeting RBS and the stem domain has been demonstrated by a multidomain antibody composed of four physically linked camelid single-domain antibodies—three of which target the stem domain and one the RBS [163]. This multidomain antibody is able confer “universal” in vivo protection against both influenza A and B viruses [163]. Furthermore, the recent development of a “mosaic” nanoparticle that co-displays HAs from multiple subtypes provides possibilities to induce such a polyclonal bnAb response [164].

## 6. Concluding Remarks

Our understanding of HA biology has advanced relentlessly every year since the first HA structure was reported in 1981. However, new unknowns in the structural biology of HA emerge as influenza viruses continues to evolve, new subtypes are found, and new zoonotic viruses enter the human population. For example, accumulation of natural mutations in the HA RBS has revealed unexpected changes in the receptor-binding modes during H3N2 evolution and motivated greater understanding of how the sialic acid receptor can continue to engage to an ever-changing binding site. In addition, discovery of the initial human bnAbs to the HA stem has inspired the discovery of new epitopes in the HA targeted by different families of bnAbs. The elucidation of how bnAbs target neutralizing epitopes on the HA has further galvanized efforts to design a variety of different classes of therapeutic candidates against the HA [142,143,144,146,147]. Such therapeutics could prevent influenza entry and infection compared to ameliorating infection as for drugs like Tamiflu and Relenza [165]. Recent advances in cryo-EM have greatly complemented X-ray crystallography and enhanced the ability to investigate full-length HA embedded in micelles or membranes [60,66]. Thus, many of the new as well as perennial unanswered questions can now begin to be addressed. In addition, the neuraminidase (NA) is also undergoing its own reincarnation from the initial antibody work in the 1960s [166] and first structures in the 1980s [167,168,169]. NA has been a neglected target on influenza virus [170] but is now undergoing a renaissance for vaccine design [8]. Structural biology of HA, as well as NA, will therefore remain a key component of influenza research until influenza virus ceases to be a global health concern, which is not yet on the horizon. We have experienced the wrath of the SARS-CoV-2 pandemic in 2020 and do not want to also experience an influenza pandemic like 1918 H1N1. Thus, effective utilization of the available and emerging structural information on HA and NA needs not only to continue to be developed but put into practice through licensed universal vaccines and therapeutics.
